# The impact of self-efficacy and sense of coherence on hand disability during the first year after distal radius fracture surgery—a prospective cohort study

**DOI:** 10.1186/s13018-026-07114-6

**Published:** 2026-07-30

**Authors:** S. Svärding, C. Mellstrand Navarro, O. Sköldenberg, M. Björk, N. Brodin

**Affiliations:** 1 Division of Orthopaedics, Danderyd’s Hospital, Stockholm, Sweden; 2 Division of Orthopaedics, Department of Clinical Sciences, Danderyd’s Hospital, Karolinska Institutet, Stockholm, Sweden; 3 Department of Clinical Science and Education, Karolinska Institutet, Södersjukhuset, Stockholm, Sweden; 4 Department of Health, Medicine and Caring Sciences, Pain and Rehabilitation Center, Linköping University, Linkoping, Sweden; 5 Division of Physiotherapy, Department of Neurobiology, Care Sciences and Society, Karolinska Institutet, Huddinge, Sweden

**Keywords:** Distal radius fracture, Hand function, Disability, Sense of coherence, Self-efficacy, Psychological factors, Rehabilitation

## Abstract

**Background:**

Distal radius fractures are among the most common surgically treated fractures in adults. While many patients recover well after surgery, a substantial number continue to experience pain, stiffness, and limitations in hand function that affect daily life and well-being. These difficulties are not always explained by physical healing alone. Psychological responses such as worry, low motivation, or reduced confidence in recovery are often observed in clinical encounters. Two psychological constructs, sense of coherence and self-efficacy, have been associated with outcomes in musculoskeletal rehabilitation. However, their contribution to recovery after distal radius fracture has not been fully established.

This study aimed to explore how sense of coherence and self-efficacy are associated with hand disability during the first year after surgical treatment.

**Methods:**

This prospective cohort study included adults (18–74 years) with surgically treated distal radius fractures. Data were collected at cast removal and at three months, six months, and one year post-surgery. Outcome measures included hand disability (DASH), sense of coherence (SOC-13), task-specific self-efficacy, pain, grip strength, and range of motion. Nonparametric statistics were used. Group differences were analyzed with Mann–Whitney U and chi-squared tests. Logistic regression explored associations between psychological factors and disability.

**Results:**

74 patients completed the three-month follow-up, and 61 remained at one year. Sense of coherence scores were stable and not associated with disability at any time point. Self-efficacy was generally high. At six months, higher self-efficacy for goal fulfilment was associated with lower disability, but no associations were found at three months or one year. Median DASH scores improved from 12 at three months to 2 at one year. Pain at rest was normalized by one year, while pain during activity remained. Grip strength and volar flexion had not fully recovered at 12 months. Supination, pronation, and radial/ulnar deviation returned to near normal by six months.

**Conclusion:**

Neither sense of coherence nor self-efficacy consistently predicted outcomes after a surgically treated distal radius fracture across all time points. However, our exploratory results contribute to the overall understanding of the multifactorial nature of recovery in this patient population.

## Background

Distal radius fractures (DRF) are the most common fractures in adults, accounting for approximately 18% of all fractures[1], with an incidence of 20–55 per 10,000 person-years[2–6]. While many patients recover well, a considerable proportion reports persistent symptoms such as pain, stiffness, and reduced grip strength[7, 8], which may impair daily functioning[9, 10]. Around 15–20% of patients continue to experience pain or moderate-to-severe disability even one year post-fracture[11–14]. These limitations affect not only physical performance but also social participation, emotional well-being, and independence[15, 16]. Recovery is not solely determined by physical healing. According to the International Classification of Functioning, Disability and Health (ICF), disability results from the interaction of health conditions with personal and contextual factors[17]. High levels of anxiety and pain catastrophizing have been linked to slower recovery, more pain, and greater disability after DRF[18, 19], and depression has been reported to be associated with persistent pain and disability after DRF[20, 21]. In the clinical encounter, patients may wonder if their symptoms are normal or worry about whether they can handle the pain and challenges of rehabilitation. Even when healing and rehabilitation are going as expected, some patients lose confidence or struggle to stay motivated. These behaviors are often not linked to the physiological components and the healing process of the DRF but can rather be explained by psychological aspects. The psychological constructs of sense of coherence and self-efficacy may help explain these responses. Sense of coherence reflects a person’s ability to perceive life as comprehensible, manageable, and meaningful, particularly under stress[22, 23], while self-efficacy refers to the belief in one’s ability to manage tasks and future challenges[24]. Sense of coherence has been associated with health outcomes in patients with major hand injuries[25, 26], in broader orthopedic populations[27], and more recently in distal radius fractures[28]. Self-efficacy has been linked to recovery after distal radius fracture[19, 29]. Previous studies investigating psychological factors after distal radius fracture have included both surgically and non-surgically treated patients[19, 30], whereas some have focused specifically on surgically treated cohorts[29]. Differences in injury severity, treatment burden, and rehabilitation demands between these groups may influence recovery and the role of psychological factors. The extent to which sense of coherence and self-efficacy can predict recovery trajectories or prolonged disability after surgically treated DRF remains unclear. This knowledge gap limits the ability to identify patients at risk of poor outcomes and to tailor rehabilitation strategies accordingly. The aim of this study was to examine the association between sense of coherence, self-efficacy, and hand disability within the first year following surgical treatment for DRF.

## Methods

### Study design

This study is a prospective observational cohort study conducted at Danderyd’s Hospital, a second-level trauma centre in Stockholm, Sweden, serving a population of approximately 600,000 individuals.

### Patients and eligibility criteria

**Fig. 1:**
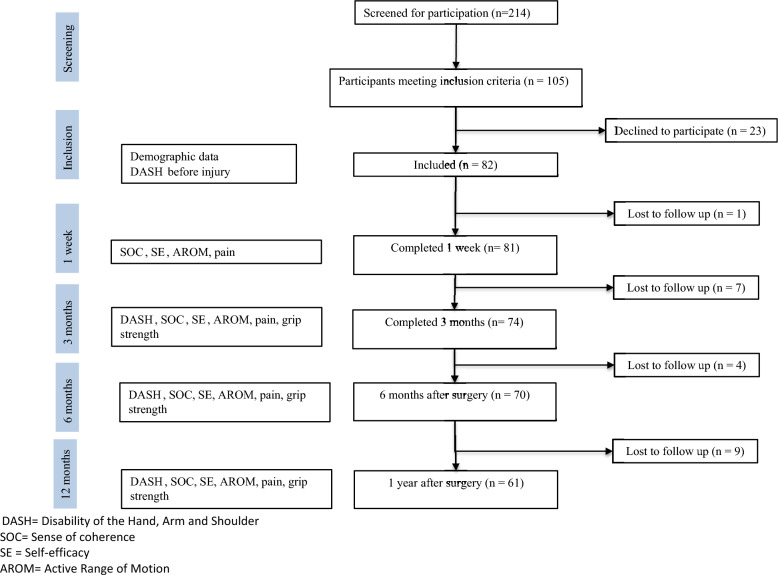
Flow chart of study investigating sense of coherence, self-efficacy, and hand and wrist function after distal radius fracture surgery

Inclusion criteria were (a) patients with a distal radius fracture surgically treated with a volar plate, (b) adult patients (aged 18–74 years at the time of injury), and (c) understanding and speaking Swedish. Exclusion criteria were: (a) other injuries or diagnoses affecting the upper extremity, (b) refractured distal radius fracture, (c) bilateral distal radius fracture, and (d) cognitive impairment. Eligible participants were invited to participate in the study during a scheduled outpatient clinic visit at the rehabilitation department, at the same time as cast removal two to five weeks post-surgery. Study information was provided verbally and in writing to facilitate informed consent and enrolment. All participants provided written consent. Recruitment took place between March 7th, 2016, and February 2nd, 2017. Out of the 105 patients who met the inclusion criteria, 23 declined participation (Fig. 1).

### Procedure

Data was collected by five occupational therapists. Their experience with hand therapy was a median of nine years. All five therapists trained together beforehand to ensure accuracy and minimize interobserver variability, and written manuals and forms were used to standardize the study data collection process.

Type of cast immobilization and restriction time, and the American Society of Anaesthesiologists (ASA) classification were obtained from the patient’s medical records. The ASA classification is a system used to assess a patient’s preoperative physical status and associated risks. It is documented on a scale from 1 to 5, where 1 indicates a healthy patient and 5 a moribund patient not expected to survive without the operation[31]. All radiographs were assessed by an orthopaedic surgeon (SaSe), and each fracture was classified according to the AO/OTA Fracture and Dislocation Classification Compendium—2018[32].

At inclusion, approximately two weeks post-DRF surgery and concurrent with cast removal, data regarding gender, age, employment, and hand dominance were collected through a personal interview, along with data on injured hand, and injury date. At the same time, patients assessed their pre-injury hand disability in the upper extremity using the Disabilities of the Arm, Shoulder, and Hand (DASH) questionnaire[33, 34]. One week after cast removal, all patients formulated individual rehabilitation goals for the upcoming rehabilitation period using a goal-setting form developed at the clinic. Sense of coherence and self-efficacy were assessed using questionnaires, and patients’ perceived certainty in managing pain, adherence to medical advice, and goal attainment. Additionally, active range of motion in the hand and wrist, as well as pain levels, were measured.

At the three-month follow-up, sense of coherence and self-efficacy were reassessed. Goals were re-formulated in relation to the coming rehabilitation period, and active range of motion, pain, and grip strength were measured. Hand disability was assessed using the DASH. This procedure was repeated at six months and one-year post-surgery (Fig. 1).

In addition to the scheduled study visits, patients were offered additional visits if needed according to local treatment guidelines. However, the type, frequency, and content of these additional visits were not systematically documented.

### Outcome measures

The primary outcome was the association between sense of coherence and hand disability. Our secondary outcomes included self-efficacy measurements and active range of motion, pain, and grip strength during the first year.

Hand disability was investigated by the Disability of the Arm Shoulder and Hand, DASH, which is a self-administered region-specific questionnaire consisting of 30 items assessing disability and symptoms related to the arm, shoulder, and hand[33, 34]. Each item is scored on a 5-point Likert-like scale. The total DASH score ranges from 0 (no disability) to 100 (most severe disability). DASH has demonstrated good reliability and validity for assessing disability in musculoskeletal conditions affecting the upper extremities, including hand and wrist injuries[35]. The DASH has been translated and validated for use in a Swedish context[34]. It has also been shown to detect changes over time in Swedish patients undergoing treatment for upper extremity conditions[36]. Cut-off points for DASH have been described: minor disability—scores 0–10, moderate disability—scores 11–35, and major disability—scores 36–100[13, 37]. Ten points is considered the minimal clinically important difference (MCID) for DASH for longitudinal changes in orthopedic injuries[38].

Sense of coherence was assessed using the 13-item version of Antonovsky’s questionnaire[22]. The items reflect the three core components of sense of coherence: five items assess comprehensibility, four manageability, and four meaningfulness. Each item is rated on a 7-point Likert scale, resulting in a total score ranging from 13 to 91. A higher total score indicates a stronger sense of coherence[39]. The Sense of Coherence Scale – 13 items (SOC-13) is a validated short version of the original 29-item instrument (SOC-29)[39]. SOC-13 has demonstrated good reliability and validity across various languages and populations[23, 40]. Notably, Antonovsky did not propose a fixed threshold or normative cut-off for sense of coherence scores[22, 41, 42]. In practice, SOC scores are understood by comparing them to the group being studied. Antonovsky did not set fixed limits but allowed researchers to decide what is normal for different groups or situations.

Patients’ self-efficacy was collected through self-assessment of specific questions regarding self-efficacy for pain management, adherence to instructions, and goal fulfilment. The questions were designed in consultation with a behavioural science expert (IM) and constructed in accordance with Bandura’s guidelines for self-efficacy scale development[43]. The patients were asked to rate, on a scale of 0–10, how they perceived their ability to perform the three activities, respectively. 0 indicates “cannot do at all,” and 10 indicates “completely certain”.

Active Range of motion was assessed using the measurement technique suggested by the Swedish Hand Surgery Quality Register[44]. Active Range of motion of the wrist and hand was measured with a universal goniometer, by recording wrist flexion–extension, pronation-supination, and radioulnar deviation. Stiffness and/or pain in the elbow and/or shoulder during movement was documented as a dichotomous outcome yes/no.

The Jamar (Hydraulic Hand Dynamometer SH 500 i) was used to measure grip strength. Three consecutive trials were made, alternating between the unaffected and the injured hand. The average of the three measurements was documented and presented in kilograms (Ewing Fess 1992, Mathiowetz et al. 1984). The non-injured hand was also tested at baseline, for comparative purposes. To ensure reliability, all measurements were carried out in a standardized measuring position[45, 46].

An 11-point visual analogue scale (VAS, 0–10) was used to assess pain at rest and during activity. VAS for assessing pain has been investigated with good validity and reliability[47, 48].

All patients were asked to formulate four individual function and/or activity goals. These goals were used to support patients’ self-efficacy ratings for the fulfilment of personal goals during rehabilitation. The first goal was formulated for the time when the individual was not allowed to put load on the hand, usually the first two to six weeks after surgery. The second goal related to the time when the individual was allowed to put load on the hand without restrictions, usually six weeks after surgery. The third and fourth goals concerned the time points at three and six months, respectively.

## Statistical analysis

All test forms and the study protocol were encoded and stored in Research Electronic Data Capture (REDCap), a secure web-based software platform for research data collection and management[49], hosted by Karolinska Institutet. Statistical analysis was performed using IBM SPSS Statistics for Windows, Version 29.0 (IBM Corp., Armonk, NY, USA). Central tendencies of numerical descriptive data were presented with median and a corresponding interquartile range or mean and standard deviation when normal data was considered. Proportions were presented with numbers and percentages. Nonparametric statistical methods were selected for the analysis due to the ordinal level of measurement, and the presence of skewed data. Mann–Whitney U-test was used for group comparisons. Proportions were compared with Chi-squared test. Findings were considered significant at a 0,05 level. For the analysis, DASH was dichotomized into minor disability (0–10) versus moderate-major disability (11–100), self-efficacy pain, following instructions, and reaching goals were dichotomized into full self-efficacy (10) or other (0–9). Due to skewed distributions and ceiling effects, self-efficacy variables were dichotomized into scores of 10 versus 0–9. This approach has been used in previous research[50]. Univariate logistic regressions were performed for each independent variable and DASH at all time points, respectively. Results for logistic regressions were expressed as odds ratios (OR) and presented with corresponding 95% confidence intervals (95% CI), and p-values. For the primary outcome, all independent variables with significance levels below 0.20 in the univariate analyses were included in a multiple logistic regression. When testing the residuals, one outlier affected the result and was removed. Thereafter, the multiple logistic regression was recalculated. Continuous variables in the model were analysed with the lowest value as a reference.

## Ethics

The study was approved by the local Ethics Committee of Stockholm with reference numbers 2016/78-31/4, 2016/154-32, and 2018/5-31.

## Results

Of the 105 patients who met the inclusion criteria, 23 declined participation, resulting in an initial cohort of 82 patients. A total of 74 patients were assessed at the three months follow up and 61 patients completed the one-year follow-up assessment. The mean age in the initial cohort, was 58 years (SD 12). There were 24 male (age range 28–73) and 58 female (age range 19–74) participants. 33% of patients had AO classification type A for distal radius fractures (DRF), 17% had type B, 50% had type C (Table 1).

**Table 1:** Demographic and disease related data (n = 82) of study investigating sense of coherence, self-efficacy and hand and wrist function after distal radius fracture surgery

		Mean (SD)	
Age		58 (12.4)	
		n	%
Sex	Male	24	29
	Female	58	71
Highest education level, n = 75	Elementary school	4	5
	High school	25	33
	Post-secondary non-tertiary education	2	3
	University	44	59
Occupation n = 77	Working	50	65
	Retired	26	34
	Student	1	1
Injured hand	Right	37	45
	Left	45	55
Injury to dominant hand, n = 76	Yes	35	46
	No	41	54
ASA^1^-class, n = 70	Class 1	37	53
	Class 2	29	41
	Class 3	4	6
AO/OTA classification^2^	A	27	33
	B	14	17
	C	41	50

^1^ASA = American Society of Anesthesiologists, ^2^AO/OTA = AO/OTA classification = Fracture classification system by the AO Foundation and Orthopaedic Trauma Association, SD = Standard deviation

At inclusion, estimated pre-injury hand disability according to the DASH was median 1 (IQR 3, range 0–31). The median DASH at three months was 12 (IQR 13, range 0–53), at six months, the median was 5 (IQR 9, range 0–68); and at one year, the median was 2 (IQR 6, range 0–21). Measures of hand function during the first year after distal radius fracture surgery are presented in Table2.

Sense of coherence scores were relatively stable over time, with a median of 54 one week after cast removal and 57 at one year (Table 3). Three months after surgery, the median SOC score was 53. In the univariate logistic regression analysis at three months, Sense of coherence was not a statistically significant predictor of low DASH scores (Table 4). As the p-value was above 0.2, sense of coherence was not included in the multivariable model.

**Table 2:** Measures of hand function during the first year after distal radius fracture surgery

		Uninjured side		Injured side, 3 months			Injured side, 6 months			Injured side, 1 year		
		Median (IQR)	Range	Median (IQR)	Range	Comparison^1^ with uninjured side, *p*	Median (IQR)	Range	Comparison^1^ with uninjured side, *p*	Median (IQR)	Range	Comparison^1^ with uninjured side, *p*
		n = 81		n = 74			n = 70			n = 61		
Pain VAS ^2^ 0–100	At rest	0 (0)	0–30	0 (6)	0–50	< 0.001	0 (2)	0–89	< 0.001	0 (0)	0–55	0.138
	During activity	0 (0)	0–16	21 (34)	0–93	< 0.001	4.5 (28)	0–91	< 0.001	0 (15)	0–65	< 0.001
Active range of motion in degrees	Volar flexion	70 (16)	38–92	55 (20)	25–80	< 0.001	62 (16)	37–80	< 0.001	65 (16)	34–80	< 0.001
	Dorsal extension	70 (11)	50–90	55 (20)	25–80	< 0.001	60.5 (13)	45–90	< 0.001	70 (15)	44–90	0.063
	Ulnar deviation	26 (10)	11–48	20.5 (9)	8–40	< 0.001	25 (10)	10–45	0.238	28 (7)	14–42	0.041
	Radial deviation	22 (7)	10–35	18 (7)	10–35	< 0.001	20 (7)	12–40	0.599	25 (10)	10–40	< 0.001
	Supination	84 (9)	70–90	80 (14)	50–90	< 0.001	80 (5)	65–95	0.210	85 (10)	65–95	0.021
	Pronation	80 (5)	60–90	80 (9)	60–90	0.007	80 (5)	60–90	0.258	80 (5)	63–90	0.454
		**n = 66**		**n = 74**			**n = 68**			**n = 61**		
Grip strength in kPa^3^		29.34 (14)	11–59	17.7 (9)	4–46	< 0.001	21.31 (9)	3–49	< 0.001	23.67 (10)	2–51	< 0.001

^1^ = Wilcoxon signed-rank test, ^2^VAS = Visual Analogue Scale, ^3^ kPa = kilopascal, IQR = interquartile range

**Table 3:** Sense of coherence, self- efficacy, and disability according to DASH^**d**^ during the first year after distal radius fracture surgery

	1 week after cast removal		3 months		6 months		1 year	
	Median (IQR)	Range	Median (IQR)	Range	Median (IQR)	Range	Median (IQR)	Range
	n = 71		n = 65		n = 64		n = 58	
SOC^a^ (13–91)	54 (9)	24–67	53 (9)	35–68	56 (8)	31–67	57 (10.5)	36–79
	n = 79		n = 74		n = 70		n = 61	
SE^b^ Managing pain from the injured wrist (0–10)	10 (2)	2–10	10 (1)	4–10	10 (0)	6–10	10 (0)	7–10
SE^b^ Adherence to instructions the coming month (0–10)	10 (2)	1–10	9 (2)	1–10	9 (2)	1–10	10 (0)^c^	3–10
SE^b^ Fulfillment of personal goals (0–10)	9 (2)	3–10	9 (2)	5–10	9 (2)	1–10	10 (2)	5–10
	**Before injury**		**3 months**		**6 months**		**1 year**	
	**n = 73**		**n = 63**		**n = 62**		**n = 59**	
DASH^d^ (0–100)	1 (3)	0–31	12 (13)	0–53	5 (9)	0–68	2 (6)	0–21

^a^SOC = Sense of coherence, ^b^SE = Self-efficacy, ^c^n = 60, ^d^DASH = Disability of the Hand, Arm and Shoulder, IQR = interquartile range

**Table 4:** Logistic regression analysis of correlation between independent variables and DASH^c^ values at 3, 6 and 12 months after distal radius fracture surgery

Variable	Univariate 3 months			Multivariable all effects 3 months Nagelkerkes R2: 0.118			Univariate 6 months			Univariate 12 months		
	OR (95%CI)	*p*-value	n	OR (95%CI)	*p*-value	n	OR (95%CI)	*p*-value	n	OR (95%CI)	*p*-value	n
SOC^a^	0.971 (0.901–1.046)	0.381	51				0.995 (0.921–1.075)	0.949	51	0.935 (0.852–1.026)	0.322	50
SE^b^ Managing pain from the injured wrist lower than 10	Ref			Ref			Ref			Ref		
SE^b^ Managing pain from the injured wrist 10	2.250 (0.761–6.648)	0.143	58	2.006 (0.657–6.128)	0.222	58	2.900 (0,868–9.691)	0.80	59	3.452 (0.610–19.539)	0.216	57
SE^b^ Adherence to instructions the coming month lower than 10	Ref			Ref			Ref			Ref		
SE^b^ Adherence to instructions the coming month 10 or else	2.893 (0.976- 8.579)	0.054	58	2.669 (0.884–8.056)	0.081	58	2.382 (0.719–7.894)	0.154	59	0.692 (0.140–3.417)	0.712	57
SE^b^ Fulfillment of personal goals lower than 10	Ref			Ref			Ref			Ref		
SE^b^ Fulfillment of personal goals 10 or else	1.874 (0.627–5.602)	0.263	58				4.940 (0.994–24.560)	0.039	59	NA	0.131	57
Pain during activity VAS^d^ 0–100	1.019 (0,992 -1.047)	0.222	58				1.016 (0.988–1.045)	0.379	59	1.016 (0.974–1.060)	0.575	57
Age	1.010 (0.964–1.058)	0.860	59				1.036 (0.977–1.098)	0.466	61	1.063 (0.958–1.181)	0.400	59
AO/OTA^e^	0.953 (0.546–1.664)	0.865	59				1.297 (0.685–2.458)	0.415	61	0.485 (0.191–1.231)	0.167	59

^a^SOC = Sense of coherence, ^b^SE = Self-efficacy, ^c^DASH = Disability of the Hand, Arm and Shoulder, ^d^VAS = Visual Analogue Scale, ^e^AO/OTA = AO/OTA Fracture and Dislocation Classification Compendium—2018, CI = Confidence Interval, OR = Odds Ratio, NA = too few observations, Continuous variables in the model with lowest value as reference

Self-efficacy for managing pain, adherence to instructions, and fulfilling personal goals was generally high but with large variation between individuals (Table 3).

In the univariate logistic regression analysis predicting low DASH scores at three months, no statistically significant predictors were found (Table 4). In the multiple logistic regression analysis for predicting low DASH scores at three months, the best model had a coefficient of determination of 0.118 (Nagelkerke’s R^2^) and included self-efficacy for managing pain and adherence to instructions. In this model, none of the variables were statistically significant.

At six months, univariate logistic regressions identified high self-efficacy for fulfilling personal goals as a statistically significant predictor of low DASH score. Again, at one year, none of the variables were statistically significant predictors of low DASH scores (Table 4).

Measures of hand function throughout the recovery trajectory revealed that pain at rest had returned to levels comparable to the uninjured side by one year after surgery. However, pain during activity remained significantly elevated at one year. While grip strength and volar flexion had not yet reached the same levels as the uninjured side after one year, functional recovery of supination and pronation was achieved within six months after surgery, and ulnar-and radial deviation reached range of motion equal to the uninjured side by six months (Table 2).

## Discussion

In this observational cohort study of patients recovering from surgically treated distal radius fractures, we investigated the potential predictive value of sense of coherence and self-efficacy for postoperative disability. Our findings did not demonstrate any significant associations, suggesting that these psychological factors may have limited utility in identifying patients at risk of poor functional outcomes in the context of standard postoperative rehabilitation for surgically treated distal radius fractures.

In our study, sense of coherence remained stable across all follow-ups. Sense of coherence has been reported to be stable in adulthood[39, 51], which is in line with our findings. While sense of coherence can be influenced by major traumatic life events[52], the severity of the distal radius fractures in our sample was probably not perceived as a major life event by the patients. Studies on patients with severe upper limb injuries have reported associations between low sense of coherence and worse disability[25, 26]. It thus seems that the role of sense of coherence in predicting outcomes may be more important in complex injuries or life-changing conditions, while its role seems less important in distal radius fractures, which can sometimes be severe but are usually not major traumatic life events.

Our results did not identify any statistically significant association between self-efficacy domains and achieving minimal hand disability (DASH ≤ 10) at three months. Although self-efficacy for adherence to instructions showed a near-significant trend in the univariate analysis (*p* = 0.054), the effect did not remain in the multivariable model (*p* = 0.081). Interestingly, self-efficacy related to fulfilment of personal goals showed a statistically significant association with recovery at six months (*p* = 0.039), suggesting a potential role in facilitating recovery during the intermediate phase of rehabilitation (i.e., between three to six months postoperatively). However, the wide confidence interval means the results should be interpreted carefully.

Previous studies have reported the predictive value of self-efficacy in recovery after a distal radius fracture. Björk et al.[29] found that higher self-efficacy was linked to better grip strength, range of motion, and better self-rated wrist function, at three months. Jayakumar et al.[19] also reported that pain self-efficacy assessed early after injury was an important predictor of physical function and general health 6–9 months into the recovery phase. In line with those findings, Schmidt et al.[30] showed that lower self-efficacy was linked to greater long-term disability in patients with DRF. However, this association was no longer significant when controlling for the other psychological factors, like pain catastrophizing, anxiety, and depression. Taken together, these findings suggest that self-efficacy may be relevant for recovery after a distal radius fracture, particularly in the early phase of rehabilitation[19, 29]. However, in the long term, its role seems less clear, and other psychological factors, such as pain catastrophizing or anxiety, may play a more prominent role[30]. Differences in study populations may also influence the strength of the association between self-efficacy and recovery outcomes across studies. Björk et al.[29] included only surgically treated patients, whereas Jayakumar et al.[19] and Schmidt et al.[30] included both surgically and non-surgically treated patients. Differences in fracture severity, treatment demands, and rehabilitation trajectories between these groups may influence the impact of psychological factors during recovery. In our study, no strong associations between self-efficacy and hand disability were observed. One possible explanation for the lack of associations in our study is that most patients reported very high self-efficacy, creating a ceiling effect. This may have reduced the variation in scores, making it harder to detect associations. Although some variation was still observed among participants, a large proportion of patients reported the maximum self-efficacy score of 10. We categorized those with maximum confidence as one group, contrasting them with participants who scored lower. This offered a way to examine how confidence levels influence recovery, as even small doubts may affect a patient’s ability to adhere to their rehabilitation program. A similar approach has previously been applied to outcome expectations, where a maximum score (10/10) was interpreted as qualitatively distinct from lower ratings[50], supporting the relevance of this type of cut-off in motivational constructs.

Even though we did not find strong associations between sense of coherence, self-efficacy, and disability, this study still provides some valuable insights into how patients understand and perceive their situation and their self-efficacy, indicating certainty in their ability to manage pain, follow instructions, and reach goals. Most participants reported high self-efficacy, meaning they were highly certain that they could manage pain, follow instructions, and reach goals. However, some had lower certainty, particularly in their ability to achieve personal goals. Low self-efficacy in reaching personal goals assessed one week after cast removal, was significantly associated with worse recovery outcomes at six months. This suggests that self-efficacy for reaching goals may be important for early identification of patients who would benefit from support several months after surgery.

These findings relate to the concept of occupational enablement, which means supporting patients in regaining the ability to engage in meaningful daily activities, even with physical limitations[53, 54]. For example**,** for patients with lower self-efficacy, it may be beneficial to formulate short-term, achievable goals together, to acknowledge small achievements, and continuously clarify their progress in the recovery process, which may contribute to feelings of control, motivation, and certainty in their rehabilitation.

Although the primary focus of this study was on psychological factors, we also assessed clinical outcomes such as pain, range of motion, and grip strength. Pain at rest had mainly returned to normal one year after surgery, whereas pain during activity remained significantly higher than on the uninjured side. These results are consistent with earlier studies showing that many patients continue to experience pain even when grip strength and movement return to normal[7]. Mehta et al.[12] also found that pain during activity remained common one year after the injury, especially when using the hand in daily activities. This was also observed despite improvements in grip strength and range of motion. They also showed that patients with high pain early on were more likely to still have problems using their hands in daily activities one year after a distal radius fracture. These results are also consistent with those of Ydreborg et al.[55], showing that pain at rest and during activity can persist 24 months after the injury, even if strength and movement are almost back to normal after 1 year.

Several planes of motion, particularly volar flexion and radial deviation, remained significantly reduced at one year postoperatively, despite improvements over time. The study shows that patients improved in both objectively measured range of motion and self-reported pain and hand disability during the whole year following a distal radius fracture, which is in line with previous research. At one year, range of motion in the wrist was nearly equal to the uninjured side.

## Strengths and limitations

This study has several strengths that contribute to its relevance and applicability. By examining sense of coherence and three dimensions of self-efficacy, the study provides a comprehensive understanding of psychological factors influencing recovery after distal radius fractures. The focus on clinically relevant outcomes, such as achieving DASH ≤ 10, ensures the practical applicability of the results to real-world rehabilitation settings. The longitudinal design, with follow-ups at three months, six months, and one year, captures recovery trajectories over time and sheds light on the possible influence of self-efficacy and sense of coherence on outcomes at different stages. Our data are derived from routine clinical practice and reflect real-world data, which enhances the external validity of our results.

Although validated self-efficacy instruments exist, these focus on more general self-efficacy and were not considered fully suitable for the specific injury and rehabilitation context of this study. Therefore, task-specific self-efficacy questions were developed in consultation with a behavioural science expert (IM) and in accordance with Bandura’s guidelines for constructing self-efficacy scales[43]. However, these questions were not formally validated, which should be considered a limitation when interpreting the results.

One important limitation of this study is the lack of a pre-study power calculation. Without it, it is difficult to determine whether the sample size was large enough to detect significant differences in patient outcomes. In addition, analysing several variables in a relatively small group of patients may have limited statistical power, increasing the risk of Type II errors (missing true associations) and Type I errors (false positives). Patients’ dropout and incomplete questionnaires further limited the statistical power and the generalizability of the findings. This may have introduced selection bias, as patients with better recovery were less likely to attend follow-ups, thereby reducing the robustness of the analyses. Another limitation is that wrist function was evaluated using the DASH questionnaire, although patient-reported outcome measures specifically developed for wrist conditions, such as the Patient-Rated Wrist Evaluation (PRWE), might have been more appropriate and sensitive for this patient group[56].

Although the data were collected almost ten years ago, clinical practice in the rehabilitation of distal radius fractures has remained largely unchanged. Therefore, our findings remain relevant.

Further research is needed to clarify whether and how psychological factors, such as sense of coherence and self-efficacy, can help identify patients who may benefit from additional support during rehabilitation. We believe that self-efficacy remains an important construct, but it should be assessed using more sensitive instruments and multidimensional approaches to better capture its nuances and avoid ceiling effects.

## Conclusion

Neither sense of coherence nor self-efficacy consistently predicted outcomes after a surgically treated distal radius fracture across all time points. However, our exploratory results contribute to the overall understanding of the multifactorial nature of recovery in this patient population.

## Data Availability

The datasets generated during and/or analysed during the current study are available from the corresponding author on reasonable request.
